# Investigating Modifiable Factors Associated with Cognitive Decline: Insights from the UK Biobank

**DOI:** 10.3390/biomedicines13030549

**Published:** 2025-02-21

**Authors:** Xiangge Ma, Hongjian Gao, Yutong Wu, Xinyu Zhu, Shuicai Wu, Lan Lin

**Affiliations:** Department of Biomedical Engineering, College of Chemistry and Life Science, Beijing University of Technology, Beijing 100124, China; maxiangge@emails.bjut.edu.cn (X.M.); gaohongjian@bjut.edu.cn (H.G.); wyt191026@emails.bjut.edu.cn (Y.W.); zhuxyu@emails.bjut.edu.cn (X.Z.); wushuicai@bjut.edu.cn (S.W.)

**Keywords:** cognitive decline, modifiable factors, UK Biobank, cognitive reserve, neurodegenerative diseases, cognitive vulnerability

## Abstract

**Objectives**: Given the escalating global prevalence of age-related cognitive impairments, identifying modifiable factors is crucial for developing targeted interventions. **Methods**: After excluding participants with dementia and substantial missing data, 453,950 individuals from UK Biobank (UKB) were included. Cognitive decline was assessed across four cognitive domains. The top 10% exhibiting the greatest decline were categorized as the “Cognitively At-Risk Population”. Eighty-three potential factors from three categories were analyzed. Univariate and multivariate Cox proportional hazards models were employed to assess the independent and joint effects of these factors on cognitive decline. Population Attributable Fractions (PAFs) were calculated to estimate the potential impact of eliminating each risk category. **Results**: Our findings revealed a significant impact of unfavorable medical and psychiatric histories on processing speed and visual episodic memory decline (Hazard Ratio (HR) = 1.34, 95% CI: 1.20–1.51, *p* = 6.06 × 10^⁻7^; HR = 1.50, 95% CI: 1.22–1.86, *p* = 1.62 × 10^⁻4^, respectively). Furthermore, PAF analysis indicated that physiological and biochemical markers were the most critical risk category for preventing processing speed decline (PAF = 7.03%), while social and behavioral factors exerted the greatest influence on preventing visual episodic memory decline (PAF = 9.68%). Higher education, socioeconomic status, and handgrip strength emerged as protective factors, whereas high body mass index (BMI), hypertension, and depression were detrimental. **Conclusions:** By identifying this high-risk group and quantifying the impact of modifiable factors, this study provides valuable insights for developing targeted interventions to delay cognitive decline and improve public health outcomes in middle-aged and older adults.

## 1. Introduction

The dramatic increase in life expectancy is a remarkable achievement [1], yet it has also amplified age-related health challenges, particularly cognitive decline [2]. Numerous studies have documented the progressive deterioration of memory and other cognitive abilities with advancing age [3,4]. Understanding the neurobiological mechanisms underlying these cognitive changes is crucial for developing effective interventions. While aging is the primary risk factor for progressive neurodegenerative diseases like Alzheimer’s disease (AD), the neurobiological mechanisms underlying cognitive deficits during typical aging differ significantly from those observed in AD [5]. Mild cognitive impairment (MCI), affecting approximately 10% of adults over 70 [6], is a transitional stage often progressing to dementia [7,8,9]. Even in the absence of MCI or dementia, cognitive impairment can significantly impact daily life, hindering activities such as cooking, managing finances, and increasing hospitalization risk [10,11].

However, the trajectory of cognitive decline varies considerably among older adults. This heterogeneity has led to the identification of various at-risk subgroups, each with its own terminology and set of risk factors. The Cognitive Vulnerability Population refers to those who are at heightened risk for cognitive impairment as they age. Kleiman et al.’s Vulnerability Index (VI), using twelve sociodemographic, medical, and functional factors, distinguishes between typical cognition and impairment [12]. Gait speed (GS) is a promising biomarker, with reduced GS correlating with poorer cognitive performance [13,14]. Nascimento et al. found low GS in older Brazilian adults, which is indicative of cognitive vulnerability [15]. Another term that has gained attention in this context is “cognitive frailty”, a clinical syndrome characterized by the co-occurrence of cognitive impairment (not meeting dementia criteria) and physical frailty in older adults. First introduced by Paganini-Hill et al. in 2001, the term was initially applied to explore associations between deficits in executive function and AD risk factors [16]. Over time, the definition has evolved to emphasize the bidirectional interplay between declining cognitive resilience and physical frailty markers, independent of concurrent dementia [17]. Contemporary frameworks position cognitive frailty as a pre-dementia state that identifies individuals at heightened risk of progression to neurodegenerative disorders [18,19]. “Low CR (Cognitive Reserve) population” refers to individuals with limited cognitive functioning and reduced adaptability to cognitive challenges [20,21]. CR, often measured by education [22], modulates adaptability to cognitive stressors, with low CR populations facing heightened decline risks [23,24]. Physical activity has also been shown to enhance CR by improving vascular and circulatory health [25], further bolstering the brain’s resilience to cognitive challenges. Finally, the subclinical cognitive impairment population represents another highly heterogeneous group characterized by early cognitive decline not meeting dementia criteria and has a high dementia risk (18% incidence over three years). This group often presents with comorbid conditions like depression or chronic illness, complicating diagnosis and management [26]. While each of these constructs contributes to our understanding of cognitive aging, the lack of consistent terminology and the fragmented approach to factor assessment limit their translational impact.

To address this gap, we define the “Cognitively At-Risk Population” as individuals exhibiting accelerated cognitive decline, performing a systematic analysis of multifactorial risks. Leveraging the UK Biobank (UKB) cohort, we (1) evaluate eighty-three factors spanning sociodemographic, medical, and lifestyle categories; (2) develop composite risk scores to quantify joint effects of interrelated risk clusters; and (3) calculate population attributable fractions (PAFs) to prioritize modifiable factors for public health strategies. By unifying fragmented terminology and quantifying the collective impact of risk categories, this work advances precision approaches to cognitive preservation and dementia prevention.

## 2. Materials and Methods

### 2.1. Study Population

The UKB, a large-scale prospective research project [27], initiated between 2006 and 2010, enrolled over 500,000 middle-aged and older adults [28]. Specifically, the baseline cohort consisted of 502,505 participants aged 37 to 73 years. The UKB population is primarily of White British ancestry, although efforts were made to recruit individuals from diverse ethnic backgrounds. Detailed information on the demographic makeup of the UKB cohort, including socioeconomic status, education level, and lifestyle factors, is available in the UK Biobank Showcase (https://www.ukbiobank.ac.uk/showcase, accessed on 24 February 2023). Subsequent phases, including repeated data collection and imaging, followed in 2012–2013, 2014, and 2019 [29]. To ensure data quality, individuals with baseline and follow-up all-cause dementia (Field ID: 42018), with more than 20% missing data or missing follow-up covariates were excluded. Dementia diagnoses followed the International Classification of Diseases, Ninth Revision (ICD-9), and Tenth Revision (ICD-10) criteria. All participants provided written informed consent before enrollment, in accordance with the Declaration of Helsinki. Ethical approval was granted by the North West Multi-Center Research Ethics Committee (REC reference: 11/NW/03820). This analysis was conducted under UKB application number 68,382 (http://www.ukbiobank.ac.uk/register-apply/, accessed on 24 February 2023).

### 2.2. Cognitively At-Risk Population

After rigorous data screening and exclusion of participants with incomplete baseline or follow-up measurements, we conducted a comprehensive cognitive assessment focusing on four key cognitive domains [30,31]: processing speed (Field ID: 20023), verbal and numerical reasoning (Field ID: 20016), visual episodic memory (Field ID: 399), and working memory (Field ID: 4282). During each participant’s assessment center visit, cognitive function was evaluated using touchscreen-based tasks. These tests are considered reliable measures of cognitive function [32]. Processing speed was assessed using a symbol–digit matching task. Participants were presented with a series of symbols paired with digits and were required to quickly match the symbols to the corresponding digits on a touchscreen. Reaction time was recorded, with a higher reaction time indicating slower cognitive processing speed. Verbal and numerical reasoning was evaluated using a logic reasoning task, in which participants were presented with a series of logical puzzles and required to select the correct answer. A higher score, reflecting the number of correctly solved puzzles, signifies better cognitive ability in this domain. Visual episodic memory was assessed through a pair-matching task, with fewer incorrect matches indicating stronger memory function. Working memory was assessed using a digit memory task, where a higher digit span reflects better working memory capacity.

To systematically characterize cognitive trajectories, we calculated the longitudinal differences between baseline scores (collected between 2006 and 2010) and scores from three subsequent follow-up assessments (2012–2013, 2014+, and 2019+). Participants in the top 10% with the greatest decline across the three assessments were classified as the “Cognitively At-Risk Population”. This calculation methodology aligns with prior research [33]. To refine the identification of this group and ensure a manageable sample size for further analysis, we limited its representation to 10% of the total cohort. Specifically, thresholds were used to define cognitive decline in each domain. An individual was considered to have experienced cognitive decline in a given domain if they met the following criteria: an increase of at least 175 milliseconds in the processing-speed score, a decrease of at least 3 points in the verbal and numerical reasoning score, an increase of at least 5 incorrect matches in the visual episodic memory, and a decrease of at least 2 points in the working memory. These criteria were employed to accurately identify individuals exhibiting significant cognitive decline in one or more domains and potential risk for neurodegenerative disorders.

### 2.3. Modifiable Factors

Potential factors were identified from the baseline data of the UKB, with variables exhibiting a missingness rate exceeding 25% being excluded from the analysis. A total of 51 factors were retained and categorized into three distinct categories. The first category, social and behavioral factors, encompasses a wide range of psychosocial and lifestyle variables. This includes socioeconomic indicators, education level, employment status, alcohol consumption, smoking habits, physical activity, dietary patterns, sleep quality, social isolation, and loneliness, among others. The second category, physiological and biochemical markers, includes handgrip strength, systolic and diastolic blood pressure, body fat percentage, trunk fat percentage, estimated glomerular filtration rate (eGFR), vitamin D levels, C-reactive protein, albumin, insulin-like growth factor 1 (IGF-1), etc. The third category, medical and psychiatric histories, encompasses conditions such as stroke, hypertension, diabetes, atrial fibrillation, coronary artery disease, depression, anxiety, bipolar disorder, sleep problems, alcohol use disorder, etc.

Detailed descriptions of these factors have previously been reported [34]. Missing values in the retained variables were imputed using random forest regression to minimize potential bias. The imputation process was conducted column by column, starting with the feature with the fewest missing values. At each step, the current column was treated as the target variable, while the remaining features (including those already imputed) were used to train the model, ensuring more accurate predictions. To provide a more comprehensive assessment of individual health risks, we conducted in-depth refinement and transformation of these variables. Categorical variables were dichotomized or trichotomized as appropriate, while continuous variables were divided into tertiles. The specific categorization and calculation methods for each variable are detailed in Supplementary Materials Table S1. Ultimately, eighty-three modifiable factors were included in the subsequent analysis.

### 2.4. Statistical Analyses

To identify modifiable factors associated with cognitive decline, we employed univariate Cox proportional hazards regression models to analyze the relationships between eighty-three factors and four cognitive declines. To address the issue of multiple comparisons, we applied a Bonferroni correction, adjusting the significance threshold to 0.0006024 (0.05 divided by eighty-three tests). All association analyses were adjusted for baseline age and sex. The proportional hazards assumption of the Cox model was verified using Schoenfeld residuals, with variables meeting this assumption at *p* > 0.001. Stratified analyses were conducted based on baseline age (<65 years vs. ≥65 years), sex (male vs. female), and follow-up duration (≥5 years vs. ≥10 years). Age and sex were consistently included as covariates in subgroup analyses where applicable.

Modifiable factors identified as significant in the univariate Cox analysis that met the proportional hazards assumption were retained for further analysis. Multicollinearity among the factors was assessed using the variance inflation factor (VIF), with all factors showing VIF values below 10 (Supplementary Materials Table S2). To maintain consistency in the direction of associations between modifiable factors and cognitive declines, favorable factors (HR < 1) identified in the univariate Cox analysis were reverse-coded to represent adverse factors. The retained risk factors were categorized into three categories: social and behavioral factors, physiological and biochemical markers, and medical and psychiatric histories. However, due to the limited number of significant risk factors for language and numerical reasoning and working memory, these categories were not further categorized.

For cognitive decline outcomes with risk factors that showed statistically significant associations after Bonferroni correction, a multivariable Cox model was developed for each category, adjusting for age and sex. The β coefficients for each risk factor were calculated, and category-specific risk scores were generated by multiplying the β coefficients by their corresponding binary variables and summing the results. Higher scores indicated greater exposure to risk factors within the category. The scores were further divided into tertiles representing favorable, intermediate, and unfavorable risk exposure within each category. However, due to highly uneven data distributions, strict tertile divisions were not always feasible. In such cases, efforts were made to minimize the discrepancies in participant numbers across categories. The final groupings are detailed in Supplementary Materials Tables S3 and S4.

To satisfy the Schoenfeld residual test and mitigate potential reverse causality, minimum follow-up times were set to ≥1000 days for processing speed and ≥3 years (1095 days) for visual declarative memory, as determined by the results of the proportional hazards test. Relationships between the three risk categories and risks of cognitive decline were initially analyzed using univariate Cox proportional hazards models, adjusting for age and sex (Model 1). Multivariate Cox proportional hazards models were subsequently employed to examine the associations between the three risk categories and risks of cognitive decline, again adjusting for the same covariates (Model 2). To evaluate interactions between the three risk categories and demographic factors, interaction terms for each category with age and sex were included in the multivariable Cox analysis, and subgroup analyses were conducted separately based on age and sex.

Finally, PAFs were calculated to estimate the proportion of cognitive decline attributable to each risk category. To simplify interpretation, three-category variables were transformed into binary variables [35]. First, moderate and favorable variables within each category were combined, and PAFs were calculated to assess the potential impact of removing adverse variables (Model 1). Second, moderate and adverse variables were grouped together, and PAFs were calculated to assess the removal of both moderate and adverse variables (Model 2). PAFs for each category were generated using the stdReg package within a univariate logistic regression framework [36].

All statistical tests were two-sided, and analyses were conducted using R version 4.3.3 and Python version 3.9.

## 3. Results

**Figure 1:**
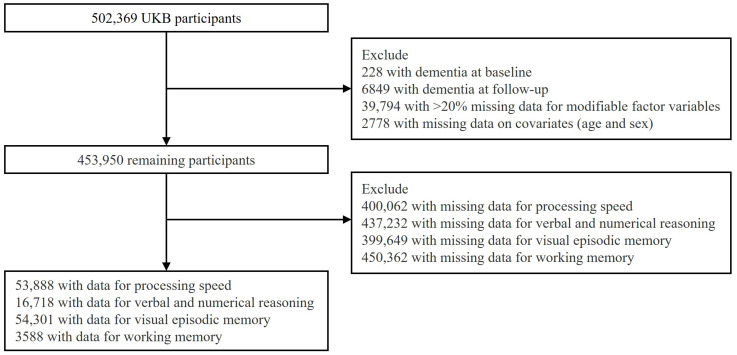
Flowchart illustrating the subject-screening process.

The participant selection process is illustrated in Figure 1. Initially, 502,369 participants from UKB were considered for inclusion. However, several exclusions were made to ensure the reliability of the analysis. First, 228 individuals with a diagnosis of dementia at baseline and 6489 individuals who developed dementia during the follow-up period were excluded. Additionally, 39,794 individuals with more than 20% missing data on modifiable factor variables and 2778 individuals with missing covariates (including age and sex) were removed. Theses exclusions resulted in a refined study cohort of 453,950 participants. To further ensure the integrity of the analysis, participants with missing baseline or follow-up data for any of the four cognitive domains were excluded. As a result, the final sample sizes varied across cognitive domains. For processing speed, 53,888 individuals (aged 55.6 ± 7.6) were included in the analysis. The sample size for verbal and numerical reasoning was 16,718 participants (aged 55.9 ± 7.6). For visual episodic memory, 54,301 participants (aged 55.6 ± 7.6) were retained, while the working memory domain had 3588 eligible participants (aged 55.0 ± 7.6) (refer to Supplementary Materials Table S5–S8 for details).

### 3.1. Modifiable Factors on Cognitive Domains

**Figure 2:**
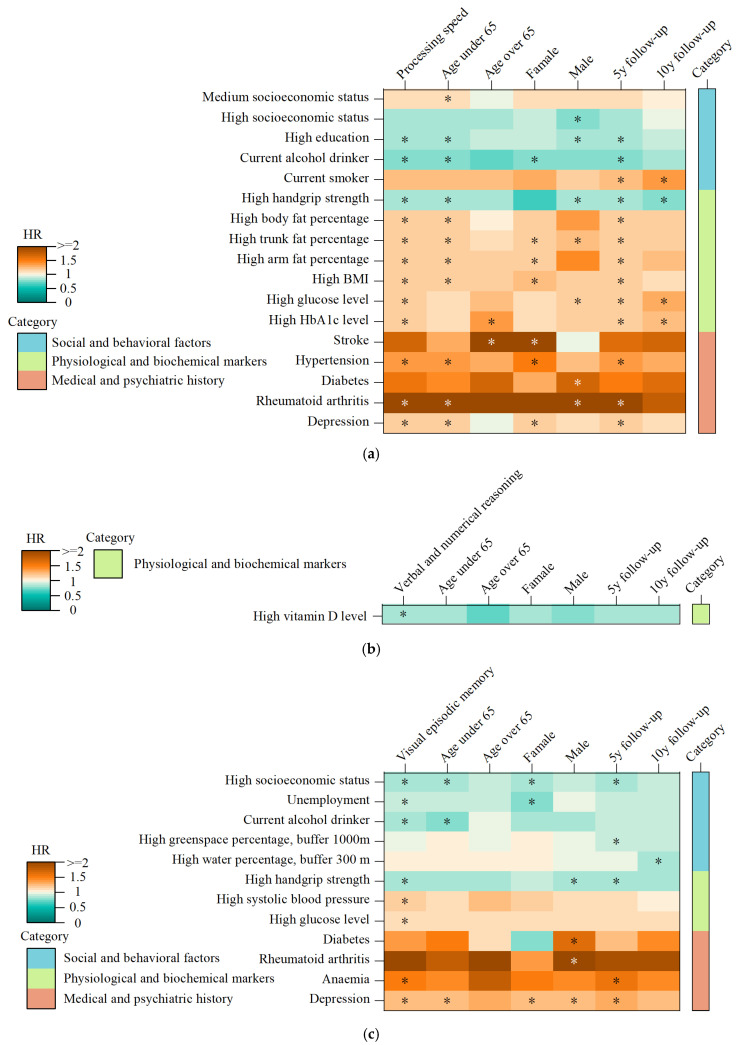
(**a**) The heatmap visualizes the significant factors associated with the risk of processing speed decline. (**b**) The heatmap visualizes the significant factors associated with the risk of verbal and numerical reasoning decline. (**c**) The heatmap visualizes the significant factors associated with the risk of visual episodic memory decline. Note: The model adjusted for baseline age and gender. The cell colors represent the effect sizes (HR) of the factors pertinent to each study group. Asterisks within the cells indicate significant associations following Bonferroni correction.

Among the eighty-three modifiable factors analyzed, twelve were found to be associated with the risk of processing-speed decline (Figure 2a). Of these, three factors exhibited protective effects, while nine factors were associated with harmful effects. Among the top five factors influencing processing speed, high education emerged as a protective factor, associated with a reduced risk of decline in processing speed (HR = 0.85, 95% CI = 0.80–0.91, *p* = 8.04 × 10⁻^7^). Conversely, four factors were linked to an increased risk of processing-speed decline: while high body mass index (BMI) was associated with a significantly higher risk (HR = 1.18, 95% CI = 1.11–1.25, *p* = 7.99 × 10⁻^8^), as was high trunk fat percentage (HR = 1.18, 95% CI = 1.11–1.26, *p* = 3.34 × 10⁻^7^). Additionally, hypertension was found to increase the risk of decline in processing speed (HR = 1.34, 95% CI = 1.19–1.50, *p* = 1.09 × 10⁻^6^), and rheumatoid arthritis exhibited a particularly strong association with increased risk (HR = 2.66, 95% CI = 1.78–3.97, *p* = 1.82 × 10⁻^6^).

In the analysis of visual episodic memory, eight factors were identified as being significantly associated with decline (Figure 2b). Of these, four factors demonstrated protective effects, while the remaining four were associated with increased risk. Among the top five factors, high socioeconomic status was associated with a reduced risk of visual episodic memory decline (HR = 0.86, 95% CI = 0.81–0.91, *p* = 1.09 × 10⁻^6^). Similarly, high handgrip strength was found to be protective against memory decline (HR = 0.86, 95% CI = 0.80–0.93, *p* = 8.98 × 10⁻^5^). Conversely, three factors were associated with a higher risk of visual episodic memory decline. Depression was one of the strongest risk factors (HR = 1.24, 95% CI = 1.16–1.33, *p* = 6.27 × 10⁻^10^), as well as high systolic blood pressure (HR = 1.13, 95% CI = 1.07–1.20, *p* = 3.41 × 10⁻^5^), and a high glucose level also contributed to increased risk (HR = 1.11, 95% CI = 1.05–1.18, *p* = 2.76 × 10⁻^4^).

In the domain of verbal and numerical reasoning, only high vitamin D levels (Figure 2c) were significantly associated with a reduced risk of decline in reasoning ability (HR = 0.81, 95% CI = 0.73–0.90, *p* = 1.85 × 10⁻^4^). No factors were identified for working memory. Furthermore, the robustness of these associations was confirmed through stratification by key demographic and follow-up variables. Age, gender, and follow-up time did not confound the observed relationships, as the results remained consistent across different subgroups (Figure 2), indicating that these associations are robust and not confounded by these variables.

### 3.2. Joint Effects of Risk Factors on Cognitive Domains

In the multi-category Cox analysis, intermediate and unfavorable risk categories were significantly associated with an increased risk of processing speed impairment compared to favorable categories (Model 2, Table 1). Specifically, intermediate and unfavorable social and behavioral factors were associated with higher risk (HR = 1.16, 1.31; 95% CI = 1.09–1.24, 1.16–1.47; *p* = 7.48 × 10⁻^6^, 8.27 × 10⁻^6^). Similarly, intermediate and unfavorable physiological and biochemical markers also increased the risk of decline in processing speed (HR = 1.13, 1.26; 95% CI = 1.05–1.22, 1.18–1.35; *p* = 9.03 × 10⁻^4^, 3.57 × 10⁻^11^). Likewise, intermediate and unfavorable medical and psychiatric histories were associated with a higher risk of processing speed impairment (HR = 1.13, 1.34; 95% CI = 1.05–1.21, 1.20–1.51; *p* = 1.32 × 10⁻^3^, 6.06 × 10⁻^7^).

Similarly, intermediate and unfavorable risk categories were found to significantly increase the risk of visual episodic memory impairment. Specifically, intermediate and unfavorable social and behavioral factors were associated with higher risk (HR = 1.11, 1.23; 95% CI = 1.04–1.19, 1.14–1.33; *p* = 1.93 × 10⁻^3^, 9.53 × 10⁻^8^). Unfavorable physiological and biochemical markers also contributed to an increased risk of visual memory decline (HR = 1.22; 95% CI = 1.13–1.32; *p* = 9.52 × 10⁻^7^). Additionally, intermediate and unfavorable medical and psychiatric histories were associated with a significantly higher risk of impairment in visual episodic memory (HR = 1.21, 1.50; 95% CI = 1.12–1.30, 1.22–1.86; *p* = 1.94 × 10⁻^7^, 1.62 × 10⁻^4^). These findings were further corroborated in the single-category Cox analysis (Model 1, Supplementary Materials Table S9).

Moreover, in the processing-speed domain, an interaction between unfavorable medical and psychiatric histories and gender was observed (Table 2). The detrimental effect was more pronounced in females (HR = 1.55, 95% CI = 1.31–1.82, *p* = 2.33 × 10⁻^7^) compared to males (HR = 1.18, 95% CI = 1.00–1.39, *p* = 4.54 × 10⁻^2^). In the domain of visual episodic memory, no interaction effects were identified (Table 3).

### 3.3. PAF Estimates for the Three Categories in Cognitive Decline Prevention

To estimate the PAF, we combined favorable and intermediate variables in a conservative scenario (Model 1). This approach revealed that 6.4% of the risk for processing speed could potentially be prevented (Table 4). The contributions to this risk reduction were distributed across various categories: social and behavioral factors accounted for 0.8%, physiological and biochemical markers accounted for 4.7%, and medical and psychiatric histories represented 0.9%. Similarly, for visual episodic memory impairment, 10.4% of risk could be prevented (Table 5). The reduction in risk was primarily driven by social and behavioral factors (7.0%), with physiological and biochemical markers contributing 3.4%. In a more extreme scenario (Model 2), where intermediate and unfavorable variables were combined to estimate the PAF after completely eliminating risk factors, the potential for risk reduction was higher. Specifically, 11.3% of processing-speed decline could be prevented. Of this, social and behavioral factors accounted for 4.3%, and physiological and biochemical markers contributed 7.0%. For visual episodic memory, the estimated risk reduction was 9.7%, with the entire contribution attributed to the elimination of adverse social and behavioral factors.

## 4. Discussion

Cognitive aging is a complex and multifactorial process that involves a combination of genetic predispositions, neurobiological mechanisms, environmental factors, lifestyle choices, and individual differences [37,38,39,40]. These factors interact in unique ways, leading to differential risk factors for cognitive decline across various cognitive domains, populations, and stages of aging. Theories such as cognitive maintenance (CM) [30,41,42] and CR [21,43] provide frameworks for understanding these processes. CM refers to the ongoing neurobiological and psychological mechanisms that support and preserve cognitive function across the lifespan. It emphasizes the importance of maintaining a healthy cognitive state through a variety of interventions and lifestyle factors. This concept posits that individuals who actively engage in cognitive and physical activities may better maintain cognitive performance by promoting neural plasticity and buffering against the effects of aging. In contrast, CR refers to the brain’s capacity to endure age-related changes or neurological insults without manifesting significant cognitive deficits. CR is thought to arise from the brain’s ability to recruit alternative neural circuits to compensate for areas that are undergoing functional decline, allowing individuals to maintain cognitive performance even in the presence of neuropathological damage. CR can be influenced by factors such as education, occupational complexity, and engagement in intellectually stimulating activities throughout life. The greater an individual’s CR, the better their brain can compensate for cognitive losses due to aging or disease. These two constructs, while distinct, are interconnected, as CM strategies can help build and strengthen CR, thus promoting resilience against cognitive decline.

Our analysis of eighty-three modifiable factors across three categories: social and behavioral, physiological and biochemical, and medical and psychiatric, revealed a significant association between these categories and an increased risk of cognitive decline. We found that most of the factors associated with cognitive decline are subsets of those previously identified as modifiable factors for dementia subtypes [34], suggesting that these factors play a role in the cognitive decline process prior to the onset of dementia. Both intermediate and unfavorable risk categories were associated with a heightened risk of cognitive decline. The potential impact of eliminating these adverse factors was substantial: a projected 11.3% reduction in processing speed decline and a 9.7% reduction in visual episodic memory decline. Among the eighty-three identified modifiable factors, twelve were associated with processing speed, and eight were linked to visual episodic memory. Importantly, some factors exhibited consistent associations across both cognitive domains. Several studies have identified a range of protective factors that influence both CR and CM. Research has demonstrated that higher levels of education [44,45] are associated with greater CR, likely reflecting the development of more complex neural networks through lifelong learning. This also contributes to CM by fostering cognitive flexibility and the ability to learn new things [46,47]. Similarly, higher socioeconomic status has been linked to increased CR [48,49] and better CM [50,51]. This may be due to better access to quality education, healthcare, and enriching environments, which promote both CR development and ongoing cognitive stimulation, such as engaging in intellectual pursuits, social interactions, and cultural activities. Physical activity is another well-established factor influencing both CR [52,53,54] and CM [55]. Regular exercise, Regular exercise not only promotes neurogenesis, improves cerebral blood flow, and enhances neuroplasticity, not only strengthens CR but also directly improves cognitive function. It enhances attention, memory, and processing speed, all crucial aspects of CM. The relationship between alcohol consumption and cognitive health is complex. While moderate alcohol consumption [56,57] within health guidelines may offer some neuroprotective benefits, excessive alcohol consumption can significantly impair cognitive function. Greater handgrip strength [58,59] has also been identified as a marker of CR. Multiple studies have established the association between handgrip strength and cognitive function. In a Korean elderly population, lower grip strength significantly increased the likelihood of cognitive impairment in both men and women [60]. Similarly, McGrath et al. [61] found that higher handgrip strength was associated with a reduced risk of future cognitive decline, while cognitive impairment predicted declines in muscle strength. Haagsma et al. [62] further demonstrated a bidirectional relationship between handgrip strength and cognitive function, highlighting the intricate interplay between physical and cognitive health. This interplay likely stems from the link between HGS and neural health, as grip strength serves as a sensitive indicator of the integrity of the motor-related nervous system [63]. Notably, greater grip strength and physical activity has been associated with larger hippocampal and frontal lobe volumes [64], suggesting that grip strength may serve as an indicator of neural function and brain health. These findings support the notion that greater handgrip strength reflects overall physical health and functional capacity, both of which are integral to maintaining CR. On the other hand, a number of factors have been associated with a reduction in CR. Smoking [65,66], has detrimental effects on brain health, including reduced blood flow and increased oxidative stress, which can impair cognitive function and diminish CR. Obesity, characterized by higher body fat percentage [67] and elevated BMI [68] has been linked to increased inflammation and insulin resistance, which can negatively impact brain function. Metabolic conditions such as diabetes, hypertension, and elevated glucose and HbA1c levels [69,70] can damage blood vessels and impair brain function, leading to a reduction in CR. Moreover, cardiovascular events, such as stroke [71,72] can cause significant brain damage, disrupting neural networks and severely impacting CR. Mental health conditions, particularly depression [73,74,75], can significantly compromise CR. Furthermore, only one factor, high vitamin D levels, was associated with a reduced risk of decline in verbal and numeric reasoning. A review on the effects of vitamin D supplementation on cognitive abilities and neurocognitive disorders in adults reported mixed results in randomized controlled trials, with one-quarter of the studies showing positive effects of vitamin D on cognition. In another study involving 210 AD patients, a 12-month vitamin D supplementation regimen improved cognitive function and reduced amyloid-beta-related biomarkers in elderly AD patients [76].

The human brain is a complex network of specialized regions, each supporting distinct cognitive domains. This compartmentalization leads to differential susceptibility to age-related decline across these domains. For example, memory function is primarily supported by the hippocampus and related structures, while language ability relies on the temporal and frontal lobes [77]. These brain regions exhibit varying vulnerability to age-related changes and neurodegenerative processes, leading to domain-specific risk factors for cognitive decline. Furthermore, individual differences in neuroplasticity and CR contribute to the differential vulnerability of cognitive domains. The impact of CR on different cognitive domains has been supported by numerous studies [78,79,80]. The concept of CM adds another layer of complexity [81,82]. Therefore, risk factors for decline might differ across domains depending on the underlying cognitive models and the neurobiological substrates involved.

Age and the length of follow-up time serve as a critical moderator in the complex trajectory of cognitive decline, significantly influencing the mechanisms and efficacy of cognitive protective factors. A growing body of research, including studies by Kraemer et al. (2019) [83] and Jin et al. (2023) [22], has consistently demonstrated that CR displays age-dependent heterogeneity in its neural protective mechanisms, with varying degrees of protective efficacy across different life stages. Sex differences further complicate this picture. While both men and women experience cognitive aging, the patterns of decline and the underlying mechanisms can differ significantly. As highlighted by Reilly (2012) and Gur & Gur (2002) [84,85], these sex differences are influenced by a complex interplay of biological and sociocultural factors. Hormonal fluctuations, particularly in women, can significantly impact cognitive trajectories [86]. The decline in estrogen levels during menopause, for example, has been associated with increased risk of AD and may contribute to specific cognitive deficits [87]. Lifestyle and health behaviors also play a crucial role. Sex-specific differences in lifestyle factors, such as physical activity, dietary habits, and stress levels, can further modulate the risk and rate of cognitive decline. For instance, women may be more susceptible to certain lifestyle-related risk factors, such as smoking or poor sleep quality, which can exacerbate age-related cognitive decline and further contribute to sex-specific risk factors.

This study, while providing valuable insights into the modifiable factors associated with cognitive decline, has several limitations. Firstly, the reliance on the UKB dataset, although extensive, may introduce selection biases. The predominantly European ancestry of the cohort may limit the generalizability of our findings to diverse populations. This could lead to an overestimation or underestimation of the effects in other ancestral groups. Future research should prioritize the inclusion of more diverse and representative cohorts from various geographical, cultural, and socioeconomic backgrounds to enhance the external validity and broader applicability of the results. Secondly, this study relied heavily on self-reported data for certain variables, such as lifestyle factors and medical history, which may be subject to recall bias and inaccuracies. Future research should incorporate objective measures, such as wearable devices for physical activity monitoring and more precise biomarker assays, to improve data accuracy. Furthermore, the cross-sectional design of the UKB data precludes definitive causal inferences. Longitudinal intervention studies are crucial to establish causal relationships between the identified modifiable factors and cognitive decline. These studies would allow for the experimental manipulation of modifiable factors and the observation of subsequent changes in cognitive function over time. Finally, while this study identified significant modifiable factors, the underlying biological mechanisms remain largely unknown. Future research should focus on elucidating the neurobiological pathways linking these modifiable factors to cognitive decline. This could involve employing neuroimaging techniques, such as MRI and PET scans, and conducting comprehensive biochemical assays to gain a more comprehensive understanding of the complex processes of cognitive aging.

## 5. Conclusions

This research significantly advances our understanding of cognitive decline in older adults by focusing on the “Cognitively At-Risk Population” within the UK Biobank dataset. By analyzing the interplay of eighty-three modifiable factors across various domains, we identified key contributors to cognitive decline, particularly medical and psychiatric histories. PAF analysis revealed the significant impact of physiological and biochemical markers on processing speed and social and behavioral factors on visual episodic memory. These findings underscore the importance of early intervention and personalized care for individuals at high risk of cognitive impairment. By identifying and supporting this vulnerable population, this research contributes to the development of targeted interventions and lifestyle modifications aimed at enhancing cognitive health and resilience, ultimately improving the quality of life and independence of older adults and reducing the burden of neurodegenerative diseases on society.

## Figures and Tables

**Table 1:** The associations between three categories and the risk of cognitive decline. (Model 2).

Categories	Processing Speed			Visual Episodic Memory		
	HR (95% CI)	*p*	*p* for Trend	HR (95% CI)	*p*	*p* for Trend
Social and behavioral factors						
Favorable	1 (reference)		***	1 (reference)		***
Intermediate	1.16 (1.09–1.24)	***		1.11 (1.04–1.19)	**	
Unfavorable	1.31 (1.16–1.47)	***		1.23 (1.14–1.33)	***	
Physiological and biochemical markers						
Favorable	1 (reference)		***	1 (reference)		***
Intermediate	1.13 (1.05–1.22)	***		1.06 (0.97–1.15)		
Unfavorable	1.26 (1.18–1.35)	***		1.22 (1.13–1.32)	***	
Medical and psychiatric histories						
Favorable	1 (reference)		***	1 (reference)		***
Intermediate	1.13 (1.05–1.21)	**		1.21 (1.12–1.30)	***	
Unfavorable	1.34 (1.20–1.51)	***		1.50 (1.22–1.86)	***	

Note: Statistical significance levels are indicated as follows: *** for *p*-values between 0 and 0.001; ** for *p*-values between 0.001 and 0.01. The precise *p*-values in Supplementary Materials Table S10. In each category, the favorable condition was used as the reference. Associations were estimated using a Cox proportional hazards model incorporating all three categories, adjusted for age and gender.

**Table 2:** Associations of the three categories with the risk of processing-speed decline in age and sex subgroups.

Categories	Age < 65		Age ≥ 65		*p* ^1^	Female		Male		*p* ^2^
	HR (95% CI)	*p*	HR (95% CI)	*p*		HR (95% CI)	*p*	HR (95% CI)	*p*	
Social and behavioral factors										
Favorable	1 (reference)		1 (reference)			1 (reference)		1 (reference)		
Intermediate	1.17 (1.09–1.25)	***	1.12 (0.96–1.32)		**	1.12 (1.03–1.23)	**	1.21 (1.09–1.33)	***	
Unfavorable	1.30 (1.14–1.48)	***	1.38 (1.05–1.82)	*		1.33 (1.15–1.54)	***	1.26 (1.04–1.52)	*	
Physiological and biochemical markers										
Favorable	1 (reference)		1 (reference)			1 (reference)		1 (reference)		
Intermediate	1.10 (1.01–1.20)	*	1.30 (1.10–1.55)	**	.	1.12 (1.00–1.26)	*	1.14 (1.03–1.26)	*	
Unfavorable	1.23 (1.14–1.33)	***	1.42 (1.19–1.69)	***		1.21 (1.11–1.32)	***	1.38 (1.23–1.56)	***	.
Medical and psychiatric history										
Favorable	1 (reference)		1 (reference)			1 (reference)		1 (reference)		
Intermediate	1.15 (1.06–1.24)	***	0.96 (0.76–1.20)		*	1.15 (1.05–1.26)	**	1.09 (0.96–1.23)		
Unfavorable	1.34 (1.17–1.54)	***	1.31 (1.05–1.62)	*		1.55 (1.31–1.82)	***	1.18 (1.00–1.39)	*	*

Note: Statistical significance levels are indicated as follows: *** for *p*-values between 0 and 0.001; ** for *p*-values between 0.001 and 0.01; * for *p*-values between 0.01 and 0.05; “.” for *p*-values between 0.05 and 0.1. The precise p-values in Supplementary Materials Table S11. In each category, the favorable condition was used as the reference. Associations were estimated using a Cox proportional hazards model incorporating all three categories, adjusted for age and gender. *p* ^1^, interaction between category and age; *p* ^2^, interaction between category and sex.

**Table 3:** Associations of the three categories with the risk of visual episodic memory decline in age and sex subgroups.

Categories	Age < 65		Age ≥ 65		*p* ^1^	Female		Male		*p* ^2^
	HR (95% CI)	*p*	HR (95% CI)	*p*		HR (95% CI)	*p*	HR (95% CI)	*p*	
Social and behavioral factors										
Favorable	1 (reference)		1 (reference)			1 (reference)		1 (reference)		
Intermediate	1.12 (1.05–1.21)	**	1.01 (0.79–1.29)			1.11 (1.01–1.23)	*	1.12 (1.02–1.23)	*	
Unfavorable	1.25 (1.15–1.36)	***	1.12 (0.91–1.38)			1.33 (1.18–1.49)	***	1.16 (1.05–1.29)	**	
Physiological and biochemical markers										
Favorable	1 (reference)		1 (reference)			1 (reference)		1 (reference)		
Intermediate	1.05 (0.96–1.16)		1.10 (0.89–1.34)			1.02 (0.78–1.35)		1.05 (0.94–1.17)		
Unfavorable	1.20 (1.10–1.31)	***	1.30 (1.09–1.55)	**		1.16 (0.87–1.54)		1.24 (1.13–1.35)	***	
Medical and psychiatric history										
Favorable	1 (reference)		1 (reference)			1 (reference)		1 (reference)		
Intermediate	1.20 (1.11–1.29)	***	1.26 (1.04–1.52)	*		1.23 (1.12–1.35)	***	1.19 (1.07–1.32)	**	
Unfavorable	1.50 (1.19–1.89)	***	1.58 (0.91–2.73)			1.53 (1.18–1.97)	**	1.48 (1.01–2.18)	*	

Note: Statistical significance levels are indicated as follows: *** for *p*-values between 0 and 0.001; ** for *p*-values between 0.001 and 0.01; * for *p*-values between 0.01 and 0.05. The precise *p*-values in Supplementary Materials Table S12. In each category, the favorable condition was used as the reference. Associations were estimated using a Cox proportional hazards model incorporating all three categories, adjusted for age and gender. *p* ^1^, interaction between category and age; *p* ^2^, interaction between category and sex.

**Table 4:** PAF of processing speed for the three categories.

Categories	Model 1		Model 2	
	PAF (95% CI)	*p*	PAF (95% CI)	*p*
Social and behavioral factors	0.008 (0.002~0.014)	4.60 × 10^−3^	0.043 (0.027~0.058)	1.74 × 10⁻^8^
Physiological and biochemical markers	0.047 (0.029~0.065)	1.21 × 10^−7^	0.070 (0.044~0.096)	1.08 × 10⁻^7^
medical and psychiatric histories	0.009 (0.003~0.015)	2.47 × 10^−3^	0.000 (−0.014~0.013)	9.46 × 10⁻^1^
Overall PAF	0.064 (0.034~0.0954)		0.113 (0.071~0.154)	

**Table 5:** PAF of visual episodic memory for the three categories.

Categories	Model 1		Model 2	
	PAF (95% CI)	*p*	PAF (95% CI)	*p*
Social and behavioral factors	0.070 (0.054~0.087)	1.81 × 10⁻^17^	0.097 (0.060~0.134)	6.94 × 10⁻^7^
Physiological and biochemical markers	0.034 (0.016~0.053)	2.21 × 10⁻^4^	−0.022 (−0.061~0.016)	2.57 × 10⁻^1^
medical and psychiatric histories	0.002 (−0.001~0.005)	2.03 × 10⁻^1^	0.006 (−0.007~0.018)	3.95 × 10⁻^1^
Overall PAF	0.104 (0.070~0.140)		0.097 (0.060~0.134)	

## Data Availability

The underlying datasets for this study originated from the UKB and are accessible through their established data access process (http://www.ukbiobank.ac.uk/register-apply/, accessed on 24 February 2023). The UKB’s Research Access Administration Team rigorously evaluates all data access requests, both academic and commercial, to ensure alignment with public health research objectives. Compliant applications are promptly approved.

## Supplementary Materials

The following supporting information can be downloaded at: https://www.mdpi.com/article/10.3390/biomedicines13030549/s1, Table S1: Definitions of modifiable factors from UK biobank; Table S2: Variance Inflation Factors(VIFs) of modifiable factors; Table S3: The distribution of data after merging risk factors associated with processing speed; Table S4: The distribution of data after merging risk factors associated with visual episodic memory; Table S5: Baseline characteristics of participants in the cognitive domain of processing speed; Table S6: Baseline characteristics of participants in the cognitive domain of verbal and numerical reasoning; Table S7: Baseline characteristics of participants in the cognitive domain of visual episodic memory; Table S8: Baseline characteristics of participants in the cognitive domain of working memory; Table S9: The associations between three categories and the risk of cognitive decline (Model 1); Table S10: The associations between three categories and the risk of cognitive decline (Model 2); Table S11: Associations of the three categories with the risk of processing speed decline in age and sex subgroups; Table S12: Associations of the three categories with the risk of visual episodic memory decline in age and sex subgroups.
